# Blended Learning Using Peer Mentoring and WhatsApp for Building Capacity of Health Workers for Strengthening Immunization Services in Kenya

**DOI:** 10.9745/GHSP-D-20-00421

**Published:** 2021-03-31

**Authors:** Iqbal Hossain, Isaac Mugoya, Lilian Muchai, Kirstin Krudwig, Nicole Davis, Lora Shimp, Vanessa Richart

**Affiliations:** a Immunization Center, JSI Research and Training Institute, Inc., Arlington, VA, USA.; b JSI Research and Training Institute, Inc., Nairobi, Kenya.; c Center for Health Information, Monitoring and Evaluation, JSI Research and Training Institute, Inc., Arlington, VA, USA.

## Abstract

Evidence from available studies suggests that peer mentoring is a useful tool to build health workers' knowledge, skills, and practices. However, there is a dearth of research on use of this method of learning in immunization programs. Although WhatsApp has been used as a networking platform among health care professionals, there is limited research on its potential contribution to improving the immunization competencies of health workers. This study showed that peer mentoring and WhatsApp networking are useful blended learning methods for need-based and individualized capacity building of health workers providing immunization services. Future research to assess the comparative cost-benefit between classroom-based training and peer mentoring (along with WhatsApp networking) will be useful.

## Abstract (precis)

Innovative learning strategies are needed to improve frontline health workers' skills for achieving immunization coverage goals—now even more important with COVID-19. Peer mentoring and WhatsApp networking are low-cost and useful blended learning methods for need-based and individualized capacity building of health workers for improving immunization services that don't disrupt the health care workers' regular work.

## Key Messages

- We piloted peer mentoring with WhatsApp for immunization capacity building of maternal and child health (MCH) nurses in the Lari and Machakos subcounties of Kenya.
- Each mentor was assigned 4 mentees and provided on-site mentoring and hands-on training for 1 year. WhatsApp networking groups supported peer-mentoring efforts.
- In both subcounties, we observed positive changes in mentees' immunization knowledge, skills, and practices.
- The WhatsApp platform improved mentees' engagement with peers and promoted discussion and learning.

## INTRODUCTION

A skilled workforce is an important determinant for a successful public health program to achieve universal health coverage.^1^ However, the health workforce is not always empowered to address current and future population health issues.^2^ Health systems in many countries often lack adequate and equitable support systems for health workers.^3^^,^^4^

Training for health care providers has traditionally been provided within the health system in the form of classroom-based methods, usually conducted at the capital or district levels.^1^^,^^5^ Immunization training has until recently been predominately via classroom-based lectures using guidance such as the World Health Organization (WHO) immunization modules.^6^ Increasingly, however, learner-centered education methods are being utilized to encourage active participation and learning, with the traditional lecture method complemented by coaching and discussion.^7^^,^^8^ Nursing education has used peer learning to help develop skills, critical thinking, and self-confidence.^9^^,^^10^ In the conventional mentoring approach, a trainee is assisted by senior staff for their professional development.^11^^,^^12^ However, relationships with peers offer important alternative benefits compared to conventionally defined mentors.^13^

Mentoring has been used widely in health programs to build health worker capacity. It has been used for quality improvement of clinical care,^14^^–^^16^ laboratory services,^17^ sexual and reproductive health and HIV/AIDS care,^18^^–^^20^ clinical nutrition,^21^ and health research.^22^ Evidence on use of mentoring for immunization capacity building of health workers is limited, as immunization training is traditionally classroom based. Standardized immunization competencies for health workers have more recently been established for workforce development.^23^ Despite the common practice of classroom-based training, peer training for routine immunization was found to improve skills and practices. ^24^

Mentoring has been used widely in health programs to build health worker capacity, but evidence on its use for immunization capacity building is limited.

With the availability of smartphones, social media applications are increasingly used for networking and learning among health care professionals.^25^^,^^26^ WhatsApp is a popular platform among health care professionals to network, communicate, and learn from each other.^27^^,^^28^ WhatsApp is easy to use, allows users to send text messages to a maximum of 256 people at once, and provides free video and image sharing.^29^^,^^30^ Evidence on the use of WhatsApp in the field of immunization is limited. However, WhatsApp use was documented during measles supplemental immunization activities for communication and coordination among health workers^31^ as well as for social networking with parents to promote seasonal influenza vaccination among young children.^32^

In this case study, we piloted peer mentoring with WhatsApp for immunization capacity building of maternal and child health (MCH) nurses in the Lari and Machakos subcounties of Kenya. We aimed to document the processes and outcomes of using these training tools for immunization capacity building of MCH nurses. We included WhatsApp as the learning platform to be used in conjunction with face-to-face peer mentoring because all MCH nurses reported already having a personal smartphone and using WhatsApp. Additionally, WhatsApp has been used by the subcounty health managers for administrative communication with health workers. This study was implemented from November 2017 to June 2019.

## PEER MENTORING PROGRAM DESCRIPTION

**FIGURE 1:**
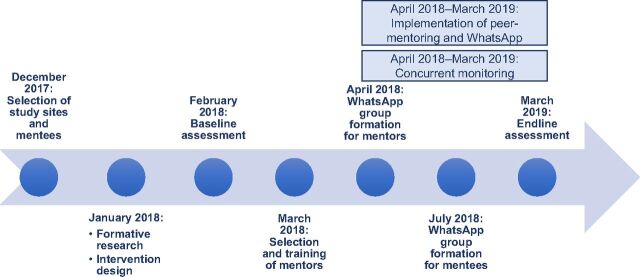
Timeline of Peer-mentoring and WhatsApp Intervention Plan for Building Capacity of Immunization Workers in 2 Subcounties in Kenya

The peer mentoring and WhatsApp study for immunization capacity building of MCH nurses consisted of the following steps: site selection, participant (mentee and mentor) selection, formative research, training of mentors, on-site mentoring, and networking using the WhatsApp platform (Figure 1).

### 1. Study Site Selection

In December 2017, using 2016 pentavalent-3 immunization coverage data extracted from the health management information system (with 60% vaccine coverage rate as the cutoff), Lari subcounty (58% coverage) in Kiambu County and Machakos subcounty (53% coverage) in Machakos County were selected. Lari and Machakos represent typical Kenyan rural and urban subcounties, respectively. In Lari, some health facilities lacked electricity and water supply, whereas in Machakos all health facilities had urban amenities (paved road, electricity, and water supply).

A total of 40 health facilities (20 in each subcounty) were selected to study the peer mentoring and WhatsApp capacity-building program. The criteria used for selecting health facilities were pentavalent-3 immunization coverage (60% vaccine coverage rate as the cutoff) and dropout rate of pentavalent vaccine from first dose to third dose (>10% as cutoff). The selected sites in Lari were 12 (60%) public health facilities, 4 (20%) faith-based organizations (FBOs), and 4 (20%) private health facilities; and in Machakos, 15 (75%) were public health facilities, 2 (10%) FBOs, and 3 (15%) private health facilities (Table 1).

**TABLE 1.** Facility and Participant Characteristics for Peer Mentoring and WhatsApp Intervention for Building Capacity in Immunization in Lari and Machako Subcounties, Kenya

Subcounty	Facility Type and OwnershipLari: N=20; Machakos: N=20			Private	Age Group	Mentor Lari: N=5; Machakos: N=5		MenteeLari: N=20; Machakos: N=20		Mentoring Visits Lari: N=19; Machakos: N=15	
	Facility Type	Public	FBO			Male	Female	Male	Female	Visits	No.
Lari	Hospital	1	1	0	<30	0	2	0	4	<4	0
	Health Center	1	1	1	30–60	0	3	3	9	4–8	0
	Dispensary	10	2	3	60+	0	0	0	4	>8	19
Total, No. (%)		12 (60%)	4 (20%)	4 (20%)		0	5 (100%)	3 (15%)	17 (85%)		19 (100%)
Machakos	Hospital	1	0	2	<30	0	0	2	2	<4	1 (7%)
	Health Center	1	0	0	30–60	1	4	2	12	4–8	6 (40%)
	Dispensary	13	2	1	60+	0	0	1	1	>8	8 (53%)
Total, No. (%)		15 (75%)	2 (10%)	3 (15%)		1(20%)	4 (80%)	5 (25%)	15 (75%)		15 (100%)

Abbreviation: FBO, faith-based organization.

### 2. Study Participant Selection (December 2017)

Study participants were selected in December 2017. In each subcounty, with the support of subcounty health officials, the MCH nurse who was responsible for providing the immunization services at each target health facility was identified and selected as a mentee. A total of 40 MCH nurses (20 in each subcounty) were selected to participate as mentees in the intervention. In Lari, 17 of the selected mentees were female (85%) and 3 (15%) were male, and in Machakos, 15 (75%) mentees were female and 5 (25%) were male (Table 1).

A total of 10 MCH nurses (5 in each subcounty) were selected to be peer mentors. Criteria for mentor selection were: (1) belonged to a health facility that was high performing in immunization, (2) willing to work as a peer mentor, and (3) had prior experience as a mentor in other health programs. All 5 mentors in Lari were female, and all but 1 mentor in Machakos were female.

### 3. Formative Research Using a Human-Centered Design Approach

In January 2018, formative research was conducted using a human-centered design (HCD) approach in 19 low- and high-performing health facilities selected conveniently (10 in Lari and 9 in Machakos). The HCD methodology was used to understand how existing capacity among MCH nurses and available communication resources could be utilized to build peer-to-peer immunization competencies and to co-design the peer mentoring and WhatsApp networking approach with the primary users. In-depth interviews were conducted with 16 MCH nurses (mentors and mentees) and supervisors in Lari and 15 MCH nurses (mentors and mentees) and supervisors in Machakos to gain insights on contextual and systemic factors, health care context, and supervision and training, structure and knowledge, and culture and communication. We asked the following key questions: how can immunization knowledge of MCH nurses be increased and barriers to performance be decreased; how can technical awareness be increased; how can dialogue be initiated among MCH nurses; and how can the cultural norms be shifted to improve adherence to immunization policy. Models of archetype MCH nurses were created to represent their needs, values, and behaviors. Based on the findings, prototyped intervention concepts of peer mentoring and use of WhatsApp were created and tested. Important takeaways from the formative research were:

1. Peer mentorship must be built on a foundation of trust. This allows nurses to feel comfortable having conversations with peers, leading to open exchanges of knowledge and skills.
2. Moderators of the WhatsApp networking groups must demonstrate an open-forum dialogue to make members feel comfortable to participate and create an environment that is conducive to free discussion while also enhancing the mentees' knowledge.

We used HCD methodology to understand how existing capacity among MCH nurses and available communication resources could be used to build peer-to-peer immunization competencies.

### 4. Mentor Training

A 2-day orientation training in March 2018 was conducted for the mentors in each subcounty to introduce the processes, steps, and challenges of peer mentoring and the use of WhatsApp networking to enhance learning beyond face-to-face mentoring. Mentor training did not include technical aspects of immunization with the assumption that they had the required immunization knowledge.

### 5. On-site Peer Mentoring

From April 2018 to March 2019, mentors met with mentees in their health facilities at least monthly. Each mentor was assigned 4 mentees. During the first mentoring visit, the mentor reviewed the baseline assessment findings with the mentee. The pair then discussed and prioritized the learning agenda and mentoring goals for the peer-mentoring sessions. The learning agenda for the mentoring sessions in both subcounties were: monitoring and data use, record keeping and reporting, problem solving, supply chain, increasing immunization coverage, cold chain management, administering vaccines, and interpersonal communication with caregivers. During subsequent mentoring visits, mentors provided hands-on training to mentees in the designated immunization technical areas. On-site peer mentoring occurred for 1 year (April 2018–March 2019).

### 6. Networking Using WhatsApp

To support peer-mentoring efforts, WhatsApp groups were formed in both subcounties. The mentors' group was formed in April 2018 and was cofacilitated by the research coordinator with support from the subcounty Expanded Program on Immunization (EPI) focal person. The mentees' group was formed in July 2018, with both mentees and mentors participating, and was facilitated by the mentors on a rotating basis (with initial support from the research coordinator).

Each mentor was provided 1,000 Kenyan Shilling (US$10) for transport costs and 500 Kenyan Shilling (US$5) per mentoring visit for lunch. In addition, each mentor was given 500 Kenyan Shilling (US$5) monthly for mobile phone airtime. Mentees were given 300 Kenyan Shilling (US$3) monthly for mobile phone airtime for participation in the WhatsApp group.

## DATA COLLECTION METHODS

### Baseline Assessment

In February 2018, baseline data collection was conducted to assess current immunization knowledge, skills, and practices of the 20 selected MCH nurses (mentees) at the 20 selected health facilities in each subcounty (total 40 in both subcounties). Data collection was done by 2 research coordinators (consultants) using an electronic data collection tool (Survey CTO) consisting of a one-on-one interview with the MCH nurse and an observation portion where the research team assessed the nurse's immunization skills and practices during a facility immunization session. The research coordinators were oriented on the survey tool by the principal investigator. The baseline assessment included: health facility type and ownership; demographic information; human resources; immunization strategy and plan; cold chain management; availability of vaccines; availability of vaccination logistics; and availability of financial resource, supervision, and immunization program monitoring.

### Concurrent Monitoring

Starting in April 2018, during each mentoring visit, mentors recorded each mentee's learning progress using a CommCare digital checklist (a mobile application). This app was used for real-time tracking of mentees' progress on the learning agenda. Additionally, postings in the WhatsApp groups of mentors and mentees were transcribed for review of the key themes of discussions within the network. Mentors shared mentoring experiences and challenges in their WhatsApp group. Mentees shared immunization technical questions, challenges, and systemic issues in their WhatsApp group.

### Endline Assessment

The endline assessment was conducted in March 2019 by the same consultants who conducted the baseline assessment. It was conducted in 34 of the 40 initially selected health facilities (19 in Lari and 15 mentees in Machakos). Six private and faith-based health facilities (1 in Lari and 5 in Machakos) dropped out of the mentoring program. A total of 6 mentees in these facilities were furloughed by the facility management due to economic conditions. All 34 remaining mentees (19 in Lari and 15 in Machakos) were assessed for immunization knowledge in the endline assessment. However, immunization skills and practices were assessed in only 30 of the mentees (17 in Lari and 13 in Machakos). The skills of 2 mentees in each sub-county could not be assessed because the facilities did not have immunization sessions on the day that the data collectors visited. The survey tool (Survey CTO) that was used in the baseline was also used in the endline assessment. In addition to the interviews for knowledge assessment and skill observations, the endline assessment also included a qualitative component in the form of focus group discussions (FGDs). Four FGDs (1 with mentors in each subcounty and 1 with 10 randomly selected mentees in each sub-county) were conducted to gather the perceptions of mentees and mentors on peer mentoring and WhatsApp as methods of learning. The key FGD questions focused upon effectiveness of peer mentoring and WhatsApp in improving the knowledge, skills, and practices of mentees for immunization services; how different peer mentoring was from other methods of capacity building; what the challenges in the process of peer mentoring were, and how useful WhatsApp was as a complement to peer mentoring for building immunization capacity.

### Ethical Review

The research protocol was reviewed by JSI Research and Training Institute institutional review board, exempted from human subject oversight, and approved by the Ministry of Health in Kenya. Written consent was obtained from all study participants before administering the study questionnaires. In addition, written consent was received from both mentors and mentees before forming WhatsApp groups for networking. Participants' names were not collected in the data collection forms, and the information they provided was kept confidential during data collection, storage, and analysis.

### Data Analysis

The CommCare checklist data were analyzed in Microsoft Excel using a scoring system. Points were given by researchers based on the observed competency of the mentees in each immunization technical area during the mentor's visit: 0 points, if the mentee was not observed to be performing in the technical area; 1 point, if the mentee needed substantial support and on-site training in the technical area; 2 points, if the mentee showed progress but still needed on-site support in the technical area; 3 points, if the mentee could perform without support in the technical area but still needed to be observed to confirm the proficiency; and 4 points, if the mentee demonstrated proficiency and could be fully independent. The scores were averaged for all technical areas and for all mentees quarterly for each subcounty.

Baseline and endline quantitative data on knowledge, skills, and practices of mentees were entered and analyzed using Microsoft Excel, and frequency tables were generated. McNemar's test was used with paired proportions of baseline and endline data to examine the improvement in mentees' immunization knowledge and skills, and practices. Risk ratio (RR) was computed with probabilities of gaps in mentees' knowledge, skills, and practices at endline and baseline assessment.

The postings in WhatsApp groups were transcribed quarterly into an electronic database to assess the participation of mentors and mentees in the groups and to identify immunization technical areas of discussion. The endline qualitative data were transcribed into an electronic database and analyzed based on emergent themes on perception of mentees and mentors regarding peer mentoring and WhatsApp networking as methods of immunization learning.

## RESULTS

### On-site Mentoring Visits

Concurrent monitoring (CommCare) data indicated that the mentoring visits varied between the 2 subcounties. In Lari, most mentees (95%) had between 9 and 12 mentoring visits and 1 received more than 12 visits over the course of the study. In Machakos, slightly more than half (53%) of mentees had between 9 and 12 visits, 40% received 5–8 visits, and 1 mentee had less than 4 visits over the course of the study (Table 1). Turnover of the mentees in some health facilities affected the number of mentoring visits received by mentees.

### Mentoring Visit Technical Content

CommCare data indicated that immunization technical content covered during mentoring visits was similar in both Lari and Machakos; however, prioritization of the content areas by the mentees differed. In Lari, prioritized content areas (ranked from highest to lowest) were: record keeping and reporting, reaching every district strategy, monitoring and use of data, vaccine supply management, increasing immunization coverage, cold chain management, interpersonal communication with caregivers, and administering vaccines. In Machakos, ranking of content areas (highest to lowest) were: recording and reporting, vaccine supply management, cold chain management, monitoring and use of data, administering vaccines, interpersonal communication with caregivers, increasing immunization coverage, and reaching every district strategy.

### Mentees' Capacity-Building Progress

**FIGURE 2:**
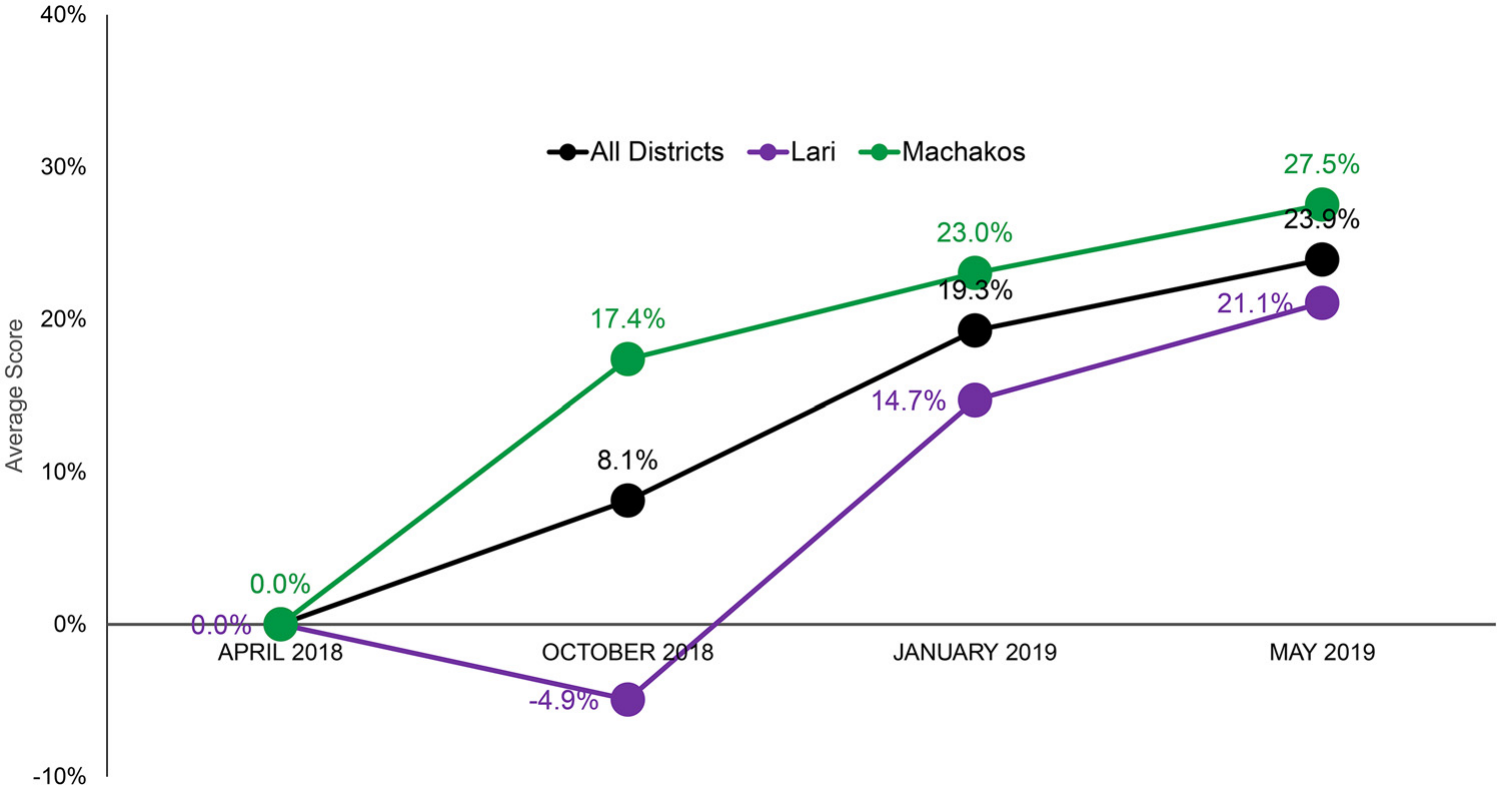
Change in Average Scores in Nurse Knowledge Across All Technical Areas of Immunization Between April 2018 and May 2019 in 2 Subcounties in Kenya

Analysis of CommCare data showed a steady increase of mentees' average scores in capacity building across all technical areas in both subcounties between the launch in April 2018 and the end of the peer mentoring program in March 2019 (Figure 2). However, the average score in Lari dipped in October 2018, since mentors were not able to complete the assessment of all mentees' progress in all technical areas by the October 2018 cutoff point.

### Networking Using WhatsApp

**FIGURE 3:**
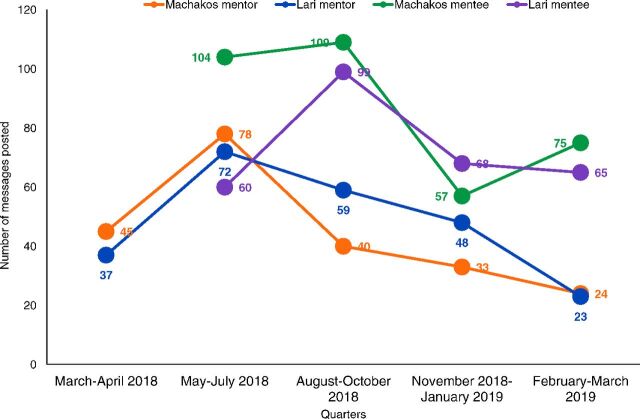
Average Number of Messages Posted per Quarter in Mentor and Mentee WhatsApp Groups in 2 Subcounties in Kenya

Analysis of transcribed WhatsApp data showed that both mentors and mentees actively participated in the WhatsApp groups and posted knowledge questions, opinions, and experiences in their respective groups. The total number of postings in the mentors' group (April 2018-March 2019) in Lari was 239 (average 20 postings/month) and in Machakos was 220 (average 18 postings/month). Posting in the mentors' groups in both subcounties dropped steadily from approximately August 2018 onward as mentors shifted to sharing their postings in the mentees' groups after their formation in July 2018 (Figure 3).

Mentors and mentees actively participated in the WhatsApp groups and posted knowledge questions, opinions, and experiences in their respective groups.

The total number of postings in the mentees' group (July 2018–March 2019) in Lari was 292 (average 32 postings/month) and in Machakos was 345 (average 38 postings/month). There was a decline in postings in the mentees' groups in November 2018–January 2019 quarter in both subcounties due to the holidays; however, postings in both the sub-counties increased in the following quarter (Figure 3). The discussion topics ranged from technical areas of administering vaccines and cold chain management to interpersonal communication with caregivers and increasing immunization coverage (Box).

BOX **Examples of Mentee Messages Shared in the WhatsApp Group on Capacity Building on Immunization**

- What information does a caregiver need to know about the child's vaccination before she leaves the immunization site?
- What is the meaning of missed opportunity of vaccination?
- What is the meaning of multidose vial, multidose vial policy, and multidose antigen vaccine?
- What is the latest time during pregnancy that a woman should take tetanus toxoid vaccine to protect her newborn baby?
- A health center received 200 doses of BCG vaccine and vaccinated 150 children. What is the wastage rate?

### Mentee Knowledge Acquisition

In Lari, comparing baseline with endline assessment data, positive changes in mentees' knowledge were found in 11 of 12 immunization technical areas (Table 2). Highly statistically significant changes in knowledge were found for: contraindication of vaccination (*P*<.0001, RR=0.13); forecasting vaccine requirement (*P*<.0001, RR=0.23); vaccination coverage rate calculation (*P*=.0039, RR=0.46); dropout rate calculation (*P*<.0001, RR=0.27); and preparation of coverage monitor chart (*P*=.0004, RR=0.37). In addition, significant change was found on knowledge of EPI target estimation (*P*=.0104, RR=0.55).

**TABLE 2.** Mentees' Immunization Knowledge Improvement From Baseline to Endline During Peer Mentoring and WhatsApp Intervention for Building Capacity in Immunization in Lari Subcounty, Kenya^a^

	Baseline, February 2018 (N=20) No. (%)	Endline, March 2019 (N=19) No. (%)	Net Percentage Gain	*P* Value p(2-tailed)	Risk Ratio
Missed opportunity of vaccination	7 (33%)	5 (26%)	−7%	.7488	1.14
Side effect of pentavalent vaccine	17 (85%)	18 (95%)	10%	.7488	0.33
Contraindications of vaccination	3 (15%)	17 (89%)	74%	<.0001	0.13
Forecasting vaccine requirement	2 (10%)	15 (79%)	69%	<.0001	0.23
Stages of vaccine vial monitor	16 (80%)	15 (84%)	4%	1.0000	1.05
Fridge tag	9 (45%)	11 (58%)	13%	.5218	0.76
Defaulter tracking	18 (90%)	18 (95%)	5%	1.0000	0.50
EPI target estimation	1 (5%)	9 (47%)	42%	.0104	0.55
Coverage rate calculation	2 (10%)	11 (58%)	48%	.0039	0.46
Dropout rate calculation	1(5%)	14 (74%)	69%	<.0001	0.27
Preparation of coverage monitor chart	2 (10%)	13 (68%)	58%	.0004	0.34
Multidose vial policy	4 (20%)	5 (26%)	6%	.7488	0.91

Abbreviation: Expanded Program on Immunization (EPI).

a Net percentage gain was calculated from the percentage of mentees who answered correctly the knowledge questions at baseline and endline. Significance (*P* value) was computed using McNemar's test with paired proportion of mentees' with correct knowledge on the topics at endline and baseline. Risk ratio was computed with probabilities of mentees' knowledge gap on the topics at endline and baseline.

In Machakos, positive changes in mentees' knowledge were found in 10 of 12 immunization technical areas (Table 3). Highly significant changes were found for: contraindication of vaccination (*P*<.0001, RR=0.15); forecasting vaccine requirement (*P*<.0001, RR=0.22); EPI target estimation (*P*=.0068, RR=0.55); coverage rate calculation (*P*<.0023, RR=0.44); dropout rate calculation (*P*<.0001, RR=0.27); and preparation of coverage monitoring chart (*P*=.0007, RR=0.36).

**TABLE 3.** Mentees' Immunization Knowledge Improvement From Baseline to Endline During Peer Mentoring and WhatsApp Intervention for Building Capacity in Immunization in Machakos Subcounty, Kenya^a^

Immunization Knowledge	Baseline, February 2018 (N=20) No. (%)	Endline, March 2019 (N=15) No. (%)	Net Percentage Gain	*P* Value (two tailed)	Risk Ratio
Missed opportunity of vaccination	7 (35%)	4 (27%)	−8%	1.0000	1.12
Side effect of pentavalent vaccine	17 (85%)	14 (93%)	8%	1.0000	0.40
Contraindications of vaccination	3 (15%)	13 (87%)	72%	<.0001	0.15
Forecasting vaccine requirement	2 (10%)	12 (80%)	70%	<.0001	0.22
Stages of vaccine vial monitor	16 (80%)	12 (80%)	0	.7353	1.0
Fridge tag	9 (45%)	9 (60%)	15%	.4990	0.72
Defaulter tracking	18 (90%)	14 (93%)	3%	.7353	0.60
EPI target estimation	1 (5%)	7 (47%)	42%	.0068	0.55
Coverage rate calculation	2 (10%)	9 (60%)	50%	.0023	0.44
Dropout calculation	1 (5%)	11 (73%)	68%	<.0001	0.27
Preparation of monitor chart	2 (10%)	10 (67%)	57%	.0007	0.36
Multi-dose vial policy	4 (20%)	4 (27%)	7%	.4990	0.91

Abbreviation: Expanded Program on Immunization (EPI).

a Net percentage gain was calculated from the percentage of mentees who answered correctly the knowledge questions at baseline and endline. Significance (*P* value) was computed using McNemar's test with paired proportion of mentees' with correct knowledge on the topics at endline and baseline. Risk ratio was computed with probabilities of mentees' knowledge gap on the topics at endline and baseline.

### Mentee Skills Acquisition

In Lari, mentees were found to have positive changes in skills and practices for 11 of 17 activities observed (Table 4). The gains were highly significant for: marking the tally sheet after each vaccination (*P*=.0085, RR=0.09); providing measles and rubella vaccination daily (*P*=.0085, RR=0.18); providing BCG vaccination daily (*P*=.0010, RR=0.43); and availability of mother and child card (*P*<.0001, RR=0.06). Changes in handwashing practices were not observed in Lari, most likely due to lack of running water or hand sanitizer in the health facilities. No changes were expected at endline on correct diluent use for BCG and measles and rubella vaccines, as this practice was found to be 100% at baseline. Negative changes for vaccine vial monitor checking before vaccination and completing the mother and child card accurately were due to new or replaced mentees who did not have adequate mentoring visits. Negative changes on availability of vaccines and vaccination materials (e.g., syringe and needles) were due to inadequate supply from sub-county offices.

**TABLE 4.** Mentees' Immunization Skill and Practice Improvement From Baseline to Endline in Peer Mentoring and WhatsApp Intervention for Building Capacity in Immunization in Lari Subcounty, Kenya^a^

Skills and Practices	Baseline, February 2018 (N=20) No. (%)	Endline, March 2019 (N=17) No. (%)	Net Percentage Gain	*P* Value (2-tailed)	Risk Ratio
Wash hands before vaccination	6 (30%)	5 (30%)	0	1.0000	1.0
Explain procedure to caregivers	12 (60%)	15 (88%)	28%	0.1884	0.27
Check Vaccine Vial Monitor (VVM) before vaccination	20 (100%)	16 (94%)	−6%	0.5108	0.00
Keep BCG, measles, and rubella diluents cold	18 (90%)	16 (94%)	4%	1.0000	0.50
Use correct diluent to reconstitute BCG, measles, and rubella	20 (100%)	17 (100%)	0	0.5108	0.00
Use nontouch injection technique	13 (65%)	15 (88%)	23%	0.3239	0.31
Dispose of used needle and syringe immediately	18 (90%)	16 (94%)	4%	1.0000	0.50
Marking each vaccination in the tally sheet	9 (45%)	16 (94%)	49%	0.0085	0.09
Complete mother and child health card accurately	20 (100%)	16 (94%)	−6%	0.5108	0.00
Complete permanent register after each vaccination	18 (90%)	16 (94%)	4%	1.0000	0.50
Correct arrangement of vaccine in the fridge	12 (60%)	16 (94%)	34%	0.1002	0.12
Temperature chart for the vaccine fridge	16 (80%)	16 (94%)	14%	0.7423	0.25
Provide measles and rubella vaccination daily	8 (40%)	15 (79%)	39%	0.0085	0.18
Provide BCG vaccination daily	1 (5%)	10 (53%)	48%	0.0010	0.43
Availability of all vaccines	19 (95%)	16 (94%)	−1%	0.7423	1.0
Availability of all vaccination materials	14 (70%)	7 (41%)	−29%	0.1002	1.93
Availability of mother and child health card	4 (20%)	16 (94%)	74%	<0.0001	0.06

a Net percentage gain was calculated from the percentage of mentees who demonstrated correct skills and practices at baseline and endline. Significance (p value) was computed using McNemar's test with paired proportion of mentees' with correct skills and practices at endline and baseline. Risk Ratio (RR) was computed with probabilities of mentees' skill and practice gaps at endline and baseline.

In Machakos, positive gains in skills and practices were found in 11 of 17 observed activities (Table 5). Highly significant changes were found for: handwashing before vaccination (*P*=.0053, RR=0.44); maintaining the temperature chart of the vaccine fridge (*P*=.0005, RR=0.33); daily BCG vaccination (*P*=.0053, RR=0.30); and providing daily measles and rubella vaccination (*P*=.0148, RR=0.32). No change was expected for completing the mother and child card accurately, as this was found to be 100% in the baseline assessment. Negative changes for checking vaccine vial monitor before vaccination and marking the tally sheet after each vaccination were due to new or replaced mentees who did not receive adequate mentoring visits. Significant negative changes on availability of vaccines and mother and child health card were due to inadequate supply from subcounty health offices.

**TABLE 5.** Mentees' Immunization Skill and Practice Improvement From Baseline to Endline in Peer Mentoring and WhatsApp Intervention for Building Capacity in Immunization in Machakos Subcounty, Kenya^a^

Skills and Practices	Baseline, February 2018 (N=20) No. (%)	Endline, March 2019(N=13) No. (%)	Net Percentage Gain	*P* Value (2-tailed)	Risk Ratio
Wash hands before vaccination	3 (15%)	8 (62%)	47%	.0053	0.44
Explain procedure to caregivers	12 (60%)	12 (92%)	32%	.2963	0.17
Check Vaccine Vial Monitor prior vaccination	20 (100%)	12 (92%)	−8%	.1637	0.00
Keep BCG, measles, and rubella diluents cold	18 (90%)	12 (92%)	2%	.4862	0.70
Use correct diluent to reconstitute BCG, measles, rubella	18 (90%)	12 (92%)	2%	.4862	0.70
Use nontouch injection technique	14 (70%)	12 (92%)	22%	.7277	0.23
Dispose of used needle and syringe immediately	19 (95%)	12 (92%)	−3%	.2963	1.40
Marking each vaccination in the tally sheet	14 (70%)	9 (69%)	−1%	.7277	1.53
Complete mother & child health card accurately	20 (100%)	13 (100%)	0	.2963	0.00
Complete permanent register after each vaccination	15 (75%)	11 (85%)	10%	1.0000	0.60
Correct arrangement of vaccine in the fridge	11 (55%)	9 (69%)	14%	.7277	0.66
Temperature chart for the vaccine fridge	4 (20%)	11 (85%)	65%	.0005	0.33
Provide measles and rubella vaccination daily	6 (30%)	10 (67%)	47%	.0148	0.32
Provide BCG vaccination daily	5 (25%)	10 (67%)	42%	.0053	0.30
Availability of all vaccines	19 (95%)	8 (62%)	−33%	.0148	7.60
Availability of all vaccination materials	16 (80%)	12 (92%)	12%	1.0000	0.35
Availability of mother and child health card	17 (85%)	4 (31%)	−54%	.0017	4.60

a Net percentage gain was calculated from the percentage of mentees who demonstrated correct skills and practices at baseline and endline. Significance (p value) was computed using McNemar's test with paired proportion of mentees' with correct skills and practices at endline and baseline. Risk Ratio (RR) was computed with probabilities of mentees' skill and practice gaps at endline and baseline.

### Perception on Using Peer Mentoring

Most mentees indicated that peer mentorship was useful in building their individualized capacity in providing routine immunizations services. Mentees reported that constant support, tracking progress, and positive feedback from mentors during peer mentoring sessions were instrumental in building knowledge and skills.

*I really benefited from mentoring and gained knowledge and skills in the technical areas of immunization—thanks to my committed mentor who was always available for me.* —Mentee in Machakos

Mentees reported that constant support, tracking progress, and positive feedback from mentors were instrumental in building their knowledge and skills.

Mentees added that peer mentoring was different from classroom-based training. Mentors addressed the individual training needs of mentees and helped them with skills development. They also noted the benefit of not needing to close the clinic for training because mentoring was done at the mentees' facilities and mentoring did not disrupt working hours.

*I am the only nurse in this health facility providing immunization services. My facility did not need to close the services for attending peer-mentoring session.* —Mentee from Lari

Upon request from the hospital management, mentees in larger health facilities also mentioned that they informally began mentoring other MCH nurses in the same facility. Mentors indicated that during mentoring visits, they were able to identify gaps in knowledge, skills, and practices that the mentees themselves were unaware of but that were necessary for providing quality routine immunization services.

*On-site mentoring allowed me to identify skill gaps in mentees and to practically demonstrate the procedures to mentees to build capacity*. —Mentor in Machakos

Mentors also indicated that initiating mentoring for peers was a challenge; however, they were able to do it through building relationships with the mentees. Challenges to mentoring included turnover of mentees and supply issues with vaccines and vaccinating materials, which prevented the mentees from putting the new knowledge and skills into practice for improving routine immunization services.

*Mentees older than me were initially ambivalent to accept me as a mentor; however, I was able to overcome this issue through building relationship with my mentees.* —Mentor in Lari

### Mentees' and Mentors' Perception of Using WhatsApp

Both mentees and mentors indicated that WhatsApp provided a platform for sharing technical questions, systemic challenges, and opinions among MCH nurses. The platform allowed increased interaction among mentees themselves and with their mentors on addressing routine immunization-related questions and challenges they encountered day-to-day in immunization service delivery.

*WhatsApp platform provided me opportunity to ask questions or share a scenario that I encountered in between the face-to-face mentoring visit of my mentor.* —Mentee in Lari

Mentees added that discussion in the WhatsApp group acted as a reminder of what they learned and as method to get further clarification of any questions and issues related to routine immunization.

*My mentor shared a lot of information during the mentoring visit, and at times, I forgot some of those. However, discussions in the WhatsApp group worked as a reminder of information my mentor provided.* —Mentee in Machakos

Interaction in the WhatsApp platform was helpful in building confidence among MCH nurses in sharing issues related to routine immunization services.

*Discussion in WhatsApp built my morale and self-confidence. It realized that I was not the only one having issues in delivering immunization services.* —Mentee in Lari

The WhatsApp platform was useful for sharing national immunization policy guidelines or other relevant reference documents to mentees on certain immunization standards.

*In case of difference of opinion among mentees in the group on certain technical areas, the group facilitator resolved the issues by providing reference from the national policy guidelines.* —Mentee in Machakos

Mentors' perception was that mentees' participation in the WhatsApp groups may have been negatively affected if the direct supervisor was nominated as the group facilitator, rather than a mentor who was not the mentee's supervisor.

## DISCUSSION

### Peer Mentoring: A Nonconventional Effective Learning Approach

The peer-mentoring program was designed to build individual immunization capacity of MCH nurses while also fostering cross learning in a less hierarchical manner.

Mentors worked with mentees to identify their individual training needs at the outset of peer mentoring, considering gaps identified during baseline assessment and challenges shared by the mentees. They worked to address gaps in mentees' knowledge and provided practical support in developing skills, and improving practices. Mentors also kept track of individual mentee's learning progress during each mentoring visit, reinforcing knowledge, skills and practices during subsequent visits and with WhatsApp discussions until the required competencies were achieved.

Mentees' positive perceptions of peer mentoring was attributed to its individualized method of learning at the facility, rather than the previous didactic lecture methods (which were conducted in classrooms rather than at the health facility). Mentees also noted that on-site peer mentoring did not disrupt their routine activities, and health facilities did not have to close facility activities for the MCH nurse to participate in the training sessions. Ndwiga et al. also reported that mentors and mentees perceived peer mentoring as an acceptable method of training.^19^ Luck et al. found that peer mentoring is a relatively cost-effective strategy requiring minimal resources and negligible disruption to clinical services.^33^

Mentees noted that on-site peer mentoring did not disrupt their routine activities.

Peer training was found to be a cost-effective method for increasing immunization coverage in health centers.^25^ However, the main objectives of our study were to document the processes and outcomes in terms of knowledge, skills/practices improvement with peer mentoring and WhatsApp. The increases in immunization coverage that arose from improved knowledge, skills, and practices could not be documented credibly in this study as stock-out of vaccines and shortage of vaccination materials affected coverage during the implementation period. During formative research, we found that MCH nurses were less likely to reach out to their supervisors either face-to-face or electronically for support related to immunization services. This may be due to the perception of the supervisor as having a position of power that is intimidating to MCH nurses. The MCH nurse mentees were found more likely to seek help from an experienced peer in gaining new knowledge, addressing a challenge, or learning an immunization skill. Mentors were nonjudgmental with mentees, creating a nonthreatening environment for mentees' learning. Mentees noted their comfort in sharing their needs and challenges. The peer-mentoring approach is an alternative to conventional mentoring in which peers are actively involved and take responsibility for their own learning. ^13^^,^^34^

### Preparing Mentors for Mentoring Visits

Mentoring is not natural for everyone and is considered a reciprocal relationship process.^35^ As such, our training for mentors helped to prepare them for peer mentoring as an important first step. We oriented them on the processes, steps, and phases of peer mentoring; listening and feedback skills; and how to do on-the-job training and provide support for performance improvement. The 5 phases of peer mentoring are^9^: (1) seeding, time of relationship building potential; (2) opening, initiation and progression of mentoring relationship; (3) laddering, period of reciprocal interaction; (4) equalizing, mentee and mentor become equal; and (5) reframing, reflection and recognition. We trained and equipped the mentors on each phase and ways to overcome potential challenges with peer mentoring. At the seeding phase, younger mentors in our program had challenges with older mentees; however, they were able to build mentees' trust by establishing a relationship. Mentees in larger health facilities even felt empowered enough to informally start mentoring other MCH nurses.

### Factors Influencing the Success of Peer Mentoring

A key to success of peer mentoring was relationship building between mentors and mentees. To build a relationship, mentors in our program presented themselves to the mentees as a helper, not as a supervisor or monitor. They created trust and a nonthreatening environment in which mentees felt comfortable sharing their challenges, with mentors listening with empathy. They provided mentees with positive feedback and avoided critiquing mentees' faults in the immunization session in front of the caregivers. In busy clinics, mentors even provided on-site support to mentees to help the mentees finish their routine work in order to free up time for mentoring sessions. One mentee stated during an FGD:

*Peer mentoring created a conducive environment in which we were comfortable to learn and share our capacity gaps and challenges.*

A mentorship based on mutual trust and respect empowers a partnership between 2 people who have a shared set of learning objectives.^9^^,^^36^ The use of the CommCare tool was useful for real-time tracking of mentees' progress.

On-site mentoring in a real-life situation (i.e., at the mentees' place of work) resulted in mentees feeling comfortable, and mentors were able to see and understand mentees' work challenges. However, support from supervisors and facility managers are critical to plan and implement on-site peer mentoring. In our program, the subcounty managers supported the selection of mentors and mentees and communicated with the facility managers regarding the peer mentoring. Support from facility managers enabled mentors to be released once a week from their regular work to participate in the mentoring activity. To avoid any interruption of immunization work in their own facilities, mentors diligently planned the mentoring visits to be on the days when there was no immunization session in the facilities. In addition, in the larger health facilities (hospitals) where immunization sessions were usually more frequent, the facility manager deputed another MCH nurse to manage the immunization sessions to allow the mentor to go for mentoring visits. Managers at the target health facilities allowed mentees to attend the mentoring sessions. County managers' support was needed to ensure that mentors and mentees were not transferred during the peer-mentoring period. In addition, support from county and subcounty managers was required to ensure that routine immunization supplies were available; however, there were instances during our study in which staff transfers and lack of vaccines and vaccination supplies negatively affected the peer-mentoring application of knowledge and skills into practices. Similar to our findings, Ndwiga et al. found successful mentorship conditional upon facility management support, sufficient supplies, a positive work environment, and mentor selection.^19^

### WhatsApp as a Networking and Learning Platform

The use of the WhatsApp platform improved MCH nurses' engagement with peers and promoted discussion and learning through sharing challenges and experiences in providing immunization services. This was consistent with other studies on WhatsApp networking among health care professionals.^37^^,^^38^ Henson et al. found difficulty in the use of social media among older age health workers and reported it to be a limiting factor for its use in other health programs.^38^ However, we found both mentors and mentees were able to participate in the WhatsApp group discussion regardless of their age in both subcounties. The research coordinators supported participants initially for logging in, and after a while, they were comfortable using it. Consistent with Amry et al.^39^ we found that the presence of a moderator in the WhatsApp group facilitated the learning process. Johnston et al. reported that WhatsApp networking helps “flatten hierarchy” among students, residents, and experienced consultants in a clinical setting by enabling all to actively contribute to discussion without inhibition.^40^ However, we found that although the role of a group moderator was important, the inclusion of a supervisor as moderator in the mentees' group could introduce power imbalances that might hinder participation of mentor and mentees. Moderation of the mentees' groups by the mentors (who were not direct supervisors) in our study created an open and nonjudgmental environment for mentees that encouraged their active participation and comfort in posting questions in the group.

Preserving patient privacy in the WhatsApp group is important for health care professionals.^41^ Both mentors and mentees in our study complied with patient privacy during WhatsApp group discussions, neither identifying by name nor adding pictures of the clients or caregivers in the WhatsApp group.

### Institutionalization, Sustainability, and Scalability of Peer Mentoring and WhatsApp

Mentoring was considered as an integral part of the continuing education process.^20^ Consistent with the findings of Ndwiga et al.^19^ the mentees and mentors in our study perceived peer-mentoring as an effective and sustainable method of capacity building to improve immunization services. We found that some mentees informally started mentoring the peers in their own health facilities. Hale indicated that in the reframing phase of peer mentoring, the mentees gain recognition of their improved knowledge and skills from the management and peers in their work place and may serve as a mentor for their peers.^9^ The capacity of the existing and newly positioned nurses can be built and updated periodically with peer mentoring backed by WhatsApp group discussions as new vaccines and technologies are added to the immunization system.

During progress update meetings with the county/subcounty health officials, we discussed the continuation of the peer mentoring and WhatsApp groups beyond the life of the project. We shared the gain in competencies of mentees and improvement in the quality of immunization services through peer-mentoring and WhatsApp. Both counties decided to continue the peer-mentoring process and WhatsApp groups. Machakos decided to scale up the initiatives to other subcounties and also decided to use peer mentoring for capacity building of health workers for other health program (e.g., family planning) using their own funds. Both subcounties decided to increase the number of mentors graduating some of the existing mentees into mentors to expand peer mentoring in all the health facilities providing immunization services. To address the performance gaps in other health facilities, supervisors in Lari were utilizing the trained mentors to improve the immunization capacity of nurses. Manzi et al. reported that integrating trained nurse mentors into the district supervision system was instrumental for quality of care improvement through providing ongoing, on-site individual mentorship to health workers in the health facilities.^14^ The WhatsApp groups in both subcounties continued after the project phased out, and group members remained active and continued to participate in the discussions.

### Limitations

1. The small sample size of the study limited precise measurement of improvement in mentees' knowledge, skills, and practices in some immunization areas with peer mentoring and WhatsApp.
2. The turnover of mentees was a limiting factor in the study. The new and replaced mentees did not receive enough peer mentoring opportunities in all immunization technical areas identified in the learning agenda.
3. The short supply and stock-out of vaccines and vaccination materials negatively affected the practices of MCH nurses (mentees) in both subcounties.
4. The increases in immunization coverage as outcome of improved knowledge, skills, and practices of mentees was not documented in this study due to performance issues related to short supply of vaccines and vaccination materials.

## CONCLUSION

The Global Vaccine Action Plan underscored the importance of building health worker capacity to support immunization programs.^42^ Innovative learning strategies outside of formal classroom trainings are needed to improve frontline health workers' competencies for achieving immunization coverage goals that have become more important now during the COVID-19 pandemic as large gatherings for face-to-face trainings are restricted. Using peer mentoring and WhatsApp for adult learning is new in immunization programs. Evidence from this study suggests that peer mentoring and WhatsApp networking could be effective methods for improving frontline health workers' on-the-job performance in immunization at minimal cost. However, to generate further evidence, a cost-benefit study would be useful to compare peer mentoring (along with WhatsApp networking) with classroom-based training for health workers.

## Notes

**Peer Reviewed**
